# Assembly of Homochiral Magneto-Optical Dy_6_ Triangular Clusters by Fixing Carbon Dioxide in the Air

**DOI:** 10.3390/molecules29143402

**Published:** 2024-07-19

**Authors:** Cai-Ming Liu, Xiang Hao, Xi-Li Li

**Affiliations:** 1 Beijing National Laboratory for Molecular Sciences, CAS Key Laboratory of Organic Solids, Institute of Chemistry, Chinese Academy of Sciences, Beijing 100190, China; haoxiang@iccas.ac.cn; 2 School of Chemical Sciences, University of Chinese Academy of Sciences, Beijing 100049, China; 3 Henan Provincial Key Laboratory of Surface and Interface Science, Zhengzhou University of Light Industry, Zhengzhou 450002, China; lixl@zzuli.edu.cn

**Keywords:** chiral complexes, Dy(III) cluster complexes, fixation of carbon dioxide, magneto-optical Faraday effect, SHG response

## Abstract

A new hydrazone Schiff base bridging ligand (H_2_L_Schiff_ (*E*)-*N*′-((1-hydroxynaphthalen-2-yl)methylene)pyrazine-2-carbohydrazide) and *L*/*D*-proline were used to construct a pair of homochiral Dy_6_ cluster complexes, [Dy_6_(CO_3_)(*L*-Pro)_6_(L_Schiff_)_4_(HL_Schiff_)_2_]·5DMA·2H_2_O (*L*-**1**, *L*-HPro = *L*-proline; DMA = *N*,*N*-dimethylacetamide) and [Dy_6_(CO_3_)(*D*-Pro)_6_(L_Schiff_)_4_(HL_Schiff_)_2_]·5DMA·2H_2_O (*D*-**1**, *D*-HPro = *D*-proline), which show a novel triangular Dy_6_ topology. Notably, the fixation of CO_2_ in the air formed a carbonato central bridge, playing a key role in assembling *L*-**1**/*D*-**1**. Magnetic measurements revealed that *L*-**1**/*D*-**1** displays intramolecular ferromagnetic coupling and magnetic relaxation behaviours. Furthermore, *L*-**1**/*D*-**1** shows a distinct magneto-optical Faraday effect and has a second harmonic generation (SHG) response (1.0 × KDP) at room temperature. The results show that the immobilization of CO_2_ provides a novel pathway for homochiral multifunctional 4f cluster complexes.

## 1. Introduction

While carbon dioxide emissions are contributing to increasing global warming, nature’s carbon cycle tells us that fixing carbon dioxide in the air into organic compounds and molecular materials is the best solution. Especially, the fixation of carbon dioxide in the air as a structural component (such as carbonato) of functional complexes is a simple but effective method because it can be carried out automatically during the synthesis process [1]. More importantly, such functional complexes formed by the immobilization of CO_2_ are usually not available directly with carbonate salts, or their structures are different from those of the products when carbonate salts are used directly [2]. So far, there have been some reports of 4f [3,4,5,6,7,8,9,10,11,12,13], 3d [14,15,16,17,18,19], and 3d–4f complexes [20,21,22,23,24,25,26,27,28,29] containing the carbonato bridge formed by the fixation of CO_2_. Notably, the automatic fixation of CO_2_ can play a critical structurally oriented role in the construction of single-molecule magnets (SMMs) [2,4,5,6,7,8,9,10,11,12,13,20,21,22,23,24,25,26,27,28,29]. The SMMs are nanoscale molecules that exhibit magnetic bistability at blocking temperatures and are expected to be used in high-density information storage, molecular electronics, and quantum computing [30,31,32,33,34,35,36,37,38]. However, the SMMs involved in the fixation of CO_2_ are still limited to achiral complexes [2,4,5,6,7,8,9,10,11,12,13,20,21,22,23,24,25,26,27,28,29].

**Scheme 1:**
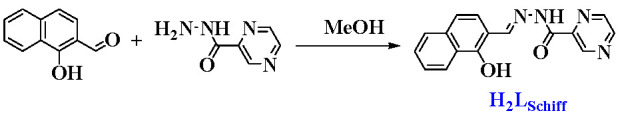
Synthesis pathway of the new hydrazone Schiff base ligand H_2_L_Schiff_.

Recently, we have been working on the construction of homochiral 3d–4f [39] and 4f [40,41,42] SMMs with Schiff base ligands to obtain strong magneto-optical Faraday effects, and such molecular materials have potential applications in magnetic-optical isolators, magnetic-optical switches, and other fields [43,44,45,46]. Furthermore, chiral SMM-induced magnetochiral dichroism (MChD) is expected to be used in the optical readout of magnetic storage data [47]. Generally, homochiral Schiff base ligands [39] and homochiral *β*-diketone ligands [40,41,42] are selected to construct such homochiral SMMs, as these two types of ligands have characteristic Cotton peaks in the circular dichroism (CD) spectra, which facilitates the study of magneto-optical Faraday effects using magnetic circular dichroism (MCD) spectra. We hope that this research will be extended to the construction of magneto-optical SMMs with simpler and cheaper chiral carboxylic acid ligands. In this paper, we adopted *L*/*D*-proline as the homochiral ligand and a new hydrazone Schiff base ligand condensed with 1-hydroxy-2-naphthaldehyde and pyrazinoic acid hydrazide as the bridging ligand (H_2_L_Schiff_, Scheme 1) to assemble a pair of Dy_6_ triangular cluster complexes, [Dy_6_(CO_3_)(*L*-Pro)_6_(L_Schiff_)_4_(HL_Schiff_)_2_]·5DMA·2H_2_O {*L*-**1**, H_2_L_Schiff_ (*E*)-*N*′-((1-hydroxynaphthalen-2-yl)methylene)pyrazine-2-carbohydrazide; *L*-HPro = *L*-proline; DMA = *N*,*N*-dimethylacetamide} and [Dy_6_(CO_3_)(*D*-Pro)_6_(L_Schiff_)_4_(HL_Schiff_)_2_]·5DMA·2H_2_O {*D*-**1**, *D*-HPro = *D*-proline}. *L*-**1**/*D*-**1** is featured by the immobilization of CO_2_ in the air during the assembly process; the corresponding carbonato ligand bridges three Dy^3+^ ions to form the central Dy_3_ triangle, which is further linked to three outer Dy^3+^ ions via the HL_Schiff_^−^/L_Schiff_^2−^ and *L*-Pro^−^ mixed bridges, generating a larger Dy_6_ triangular cluster. Such a triangular Dy_6_ topology has never been reported in carbonato-bridged Dy_6_ cluster complexes. Notably, in addition to intramolecular ferromagnetic coupling and magnetic relaxation at 0 Oe, *L*-**1**/*D*-**1** shows a magneto-optical Faraday effect and an SHG response at room temperature.

## 2. Results and Discussion

### 2.1. Synthesis and Crystal Structures

When the Dy(III) complexes are assembled with hydrazone Schiff base ligands in an alkaline solution, the automatic fixation of CO_2_ in the air can occasionally occur, and the carbonato-bridged Dy(III) cluster complexes are finally obtained [2,4,6,8,12], which include Dy_6_ complexes with topologies of the triangular prism [2,8,12] and the fusion of three capped triangular Dy_3_ motifs [4]. *L*-**1**/*D*-**1** was yielded by the reaction of 0.25 mmol of H_2_L_Schiff_, 0.25 mmol of Dy(CF_3_SO_3_)_3_, 0.25 mmol of *L*/*D*-proline, and 0.75 mmol of LiOH·H_2_O in 5 mL of DMA for 24 h at room temperature. In general, the reaction at room temperature can effectively prevent the rapid racemization of chiral complexes at high temperatures [48]. Good-quality single crystals of *L*-**1**/*D*-**1** could be obtained by recrystallization with CH_2_Cl_2_-MeOH (1:1 *v*/*v*), which crystallizes in the monoclinic C_2_ space group. If DMA is changed to DMF, the product with the same structure can also be obtained, but the lattice solvent DMA becomes DMF, and the single crystal diffraction data will be worse. Notably, the Dy(III) cluster complexes constructed with *L*/*D*-proline are very rare [49], and *L*-**1**/*D*-**1** represents the first complex constructed from the hydrazone Schiff base ligand H_2_L_Schiff_.

**Figure 1:**
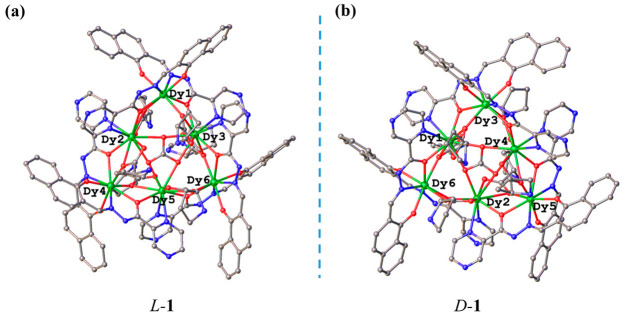
Crystal structures of *L*-**1** (**a**) and *D*-**1** (**b**). Color code: C, grey; N, blue; O, red; Dy, green. All disordered pyrrolidine rings show only one of two sets, and all H atoms and solvent molecules are omitted for clarity.

As shown in Figure 1a, the carbonato ligand formed by the automatic fixation of CO_2_ in the air acts as the structural core of *L*-**1**, which directly bridges three Dy^3+^ ions in a triangular shape in a hexadentate μ_3_-mode, with Dy…Dy separation distances of 4.793, 4.867, and 4.745 Å for Dy2-Dy3, Dy3-Dy5, and Dy2-Dy5, respectively. Such a CO_3_^2−^-bridged Dy_3_ central triangle is further linked to three additional Dy^3+^ ions through the HL_Schiff_^−^/L_Schiff_^2−^ and *L*-Pro^−^ mixed bridges, eventually forming a larger Dy_6_ triangular cluster, which contains three small outer Dy_3_ triangles. Such a triangular Dy_6_ topology is different from the triangular prism [2,8,12] and the fusion of three capped triangular Dy_3_ motifs [4] in carbonato-bridged Dy_6_ cluster complexes. The outer Dy…Dy separation distances are 3.987 Å for Dy2-Dy4, 3.930 Å for Dy5-Dy4, 3.959 Å for Dy1-Dy2, 3.906 Å for Dy1-Dy3, 3.926 Å for Dy3-Dy6, and 3.931 Å for Dy5-Dy6, which are smaller than those in the Dy_3_ central triangle (4.745–4.867 Å). Notably, the μ_3_-η^2^:η^2^:η^2^ coordination mode of the carbonato ligand in *L*-**1** is also observed in the triangular prism-like Dy_6_ cluster complexes assembled by other hydrazone Schiff base ligands [2,8,12] but is different from the μ_3_-η^2^:η^2^ coordination mode in the Dy_6_ cluster complex with a topology of the fusion of three capped triangular Dy_3_ motifs [4] and the μ_4_-η^1^:η^2^:η^1^:η^1^ coordination mode in the tub-like Dy_8_ cluster complex [2,6]. The Schiff base ligands in *L*-**1** have not only a deprotonated form (L_Schiff_^2−^) but also a protonated form (HL_Schiff_^−^); both bridge the two Dy^3+^ ions on the outer Dy-Dy edge of the outer small Dy_3_ triangle, and this Dy-Dy edge is simultaneously bridged by the carboxyl group of one *L*-Pro^−^ anion; the latter adopts a unique μ_2-_η^1^:η^2^ coordination mode in which the pyrrolidine N atom does not participate in the coordination, while it usually uses both the carboxyl O atom and the pyrrolidine N atom to participate in coordination [49].

The Dy^3+^ ions located on the central Dy_3_ triangle (Dy2, Dy3, and Dy5) are 10-coordinated (Figure 1), which are coordinated by two O atoms from the carbonato ligand, four O_carboxyl_ atoms from two *L*-Pro^−^ anions, two O_carboxyl_ atoms and two N_pyrazine_ atoms from two L_Schiff_^2−^/HL_Schiff_^−^ ligands. An accurate analysis by the Shape 2.1 software [50] revealed that the coordination configurations of these Dy^3+^ ions are the bicapped square antiprism J17 (D_4d_), and the CShM values are 2.886 for Dy2, 3.156 for Dy3, and 3.052 for Dy5 (Table S1). However, the Dy^3+^ ions located at the vertices of the large Dy_6_ triangle (Dy1, Dy4, and Dy6) are 8-coordinated (Figure 1), which are bonded by two O_carboxyl_ atoms from two *L*-Pro^−^ anions, two O_carboxyl_ atoms, two N_pyrazine_ atoms and two O_phenol_ atoms from two L_Schiff_^2−^/HL_Schiff_^−^ ligands. An accurate analysis by the Shape 2.1 software [50] showed that the coordination configurations of these three Dy^3+^ ions are the following: the biaugmented trigonal prism J50 (C_2v_) for Dy1 and Dy4, with CShM values of 2.249 and 2.679, respectively (Table S2); the biaugmented trigonal prism (C_2v_) for Dy6, with a CShM value of 2.118 (Table S2). *D*-**1** is an enantiomer of *L*-**1** and thus has a structure that is similar and mirror symmetrical to *L*-**1** (Figure 1b).

### 2.2. Magnetic Properties

**Figure 2:**
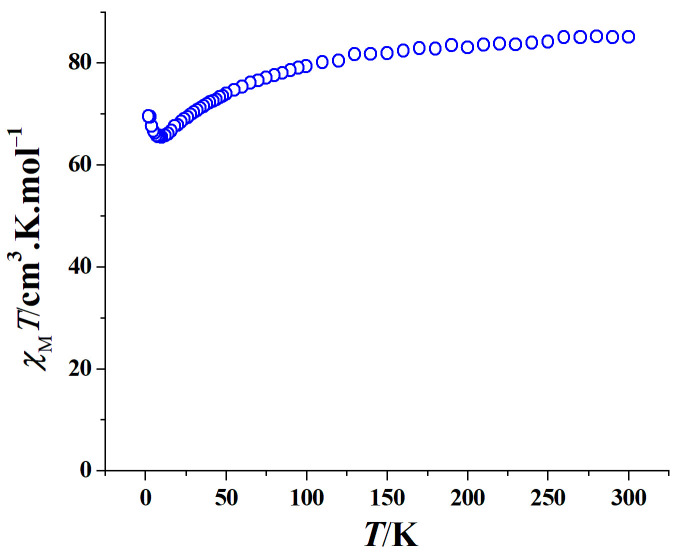
Plot of χ_M_T vs. T of *L*-**1** (H_dc_ = 1000 Oe).

The temperature-dependent DC magnetic susceptibility of *L*-**1** measured at 1000 Oe revealed that its χ_M_T value (85.10 cm^3^ K mol^−1^) at 300 K is in agreement with the theoretical value of six isolated Dy^3+^ ions (85.02 cm^3^ K mol^−1^) (Figure 2). The χ_M_T value of *L*-**1** decreases as it cools down, reaching a minimum of 65.44 cm^3^ K mol^−1^ at 10 K, and then suddenly increases to 69.55 cm^3^ K mol^−1^ at 2 K. Such a hook-like χ_M_T–T curve is the result of a combination of two main factors: the depopulation of the m_J_ levels of the Dy^3+^ ions and the intramolecular ferromagnetic coupling among the Dy^3+^ ions [12,40,42,51]. Similar intramolecular ferromagnetic interactions between Dy^3+^ ions are also present in another carbonato-bridged dysprosium (III) complex [6]. Moreover, the magnetic field-dependent magnetization measurements of *L*-**1** showed no overlap in the M–H/T curves at 2–6 K (Figure S1), indicating magnetic anisotropy.

**Figure 3:**
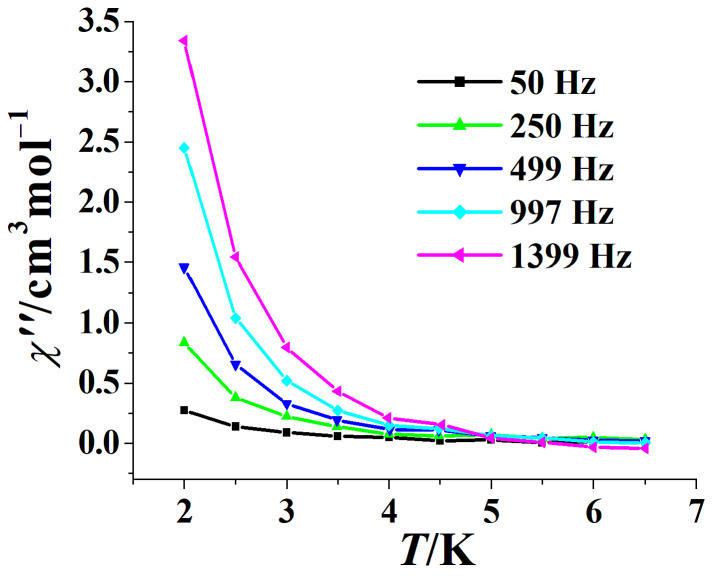
Plots of χ″ vs. T for *L*-**1** (H_dc_ = 0 Oe).

The temperature-dependent AC magnetic susceptibility of *L*-**1** was measured in the range of 50–1399 Hz at 0 Oe to investigate its SMM properties. The χ″ vs. T curves show frequency dependence, but no peaks, and the χ″ signal appears only in the lower temperature region (<5 K) (Figure 3), suggesting that the effective energy barrier value of *L*-**1** is very small. Furthermore, the χ″–T curve at 1399 Hz also shows no signs of peaking when a common DC field of 2000 Oe is applied (Figure S2), suggesting that the failure to show peaks is not caused by the quantum tunnelling effect. Such a phenomenon also occurs in the Dy_5_ cluster complexes constructed from *L*/*D*-proline [49] and other Dy(III) cluster complexes formed by the immobilized CO_2_ [5,7].

The Debye model based on Equation (1), in which ω (=2πν) represents the angular frequency [52], can generally be used to roughly estimate the U_eff_/k value and the τ_0_ value [53].

ln(χ″/χ′) = ln(ωτ_0_) + U_eff_/kT (1)

It is worth mentioning that such a fitting method is only suitable for when the χ″–T curves do not have peaks, and the U_eff_/k and τ_0_ values may be different when fitting with χ″–T curves of different frequencies [54]. The best fitting of ln(χ″/χ’)–1/T curves for *L*-**1** leads to U_eff_/k values of 6.5–8.3 K and τ_0_ values of 2.0 × 10^−7^–6.5 × 10^−7^ s (Figure S3). The U_eff_/k value of *L*-**1** is comparable with that of the hourglass-like {Dy_7_} cluster complex (~4.6 K) [53]. In Dy^3+^ multinuclear cluster complexes, the magnetic axes of different ions are difficult to regularize, and factors such as J-type exchange coupling and intramolecular and intermolecular dipole interactions are often not conducive to good SMM performance [32]; these factors are also likely to be the reason for the smaller U_eff_/k value of *L*-**1**.

### 2.3. Circular Dichroism Spectra (CD) and Magnetic Circular Dichroism Spectra (MCD)

**Figure 4:**
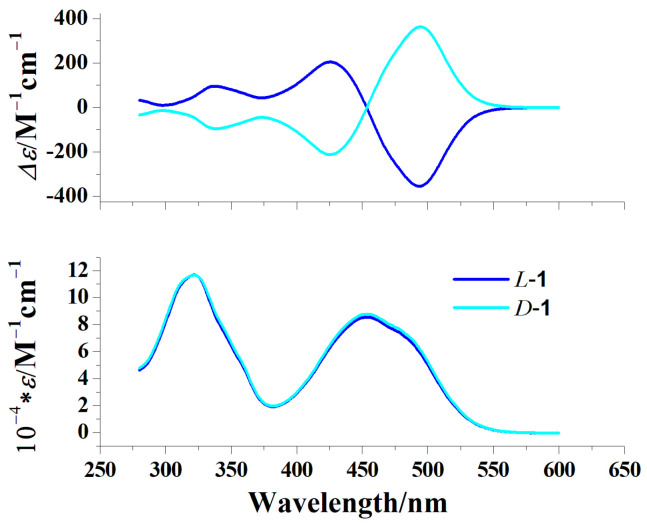
CD spectra of *L*-**1** and *D*-**1** in DMF solution (c = 0.2 gL^−1^; H = 0 T) (**above**) and UV-vis spectra of *L*-**1** and *D*-**1** in DMF solution (c = 0.2 gL^−1^; H = 0 T) (**below**).

In order to verify the properties of the enantiomers, the CD spectra of *L*-**1** and *D*-**1** in DMF solution were measured along with their UV-vis spectra (Figure 4). The UV-vis spectrum of *L*-**1** is similar to that of *D*-**1** (Figure 4, below), the strong peak at 322 nm may be ascribed to the π-π* conjugation of the naphthalene ring and the C=N bond of the hydrazone Schiff base ligand, while the strong peak at 454 nm and its shoulder peak at a higher wavelength are attributed to the n-π* transition of the C=N bond of the hydrazone Schiff base ligand [39,41,42,45,46]. In contrast, the UV-vis spectrum of the H_2_L_Schiff_ ligand in DMF solution can clearly distinguish the absorption peaks of the pyrazine group (323 nm), 1-naphthol group (338 nm) [55], and the π-π* conjugation of the naphthalene ring and the C=N bond (373 nm), while the absorption peak of the n-π* transition of the C=N bond at 475 nm is very weak (Figure S4). The CD spectra of *L*-**1** and *D*-**1** exhibit excellent mirror symmetry, confirming their enantiomeric properties (Figure 4, above). The Cotton peak at 298 nm is assigned to the π-π* transitions of the conjugated group in the hydrazone Schiff base ligand, while the Cotton peaks at 374 nm and 428 nm are caused by the π-π* and n-π* transitions of the C=N chromophore, respectively [42,45,46].

**Figure 5:**
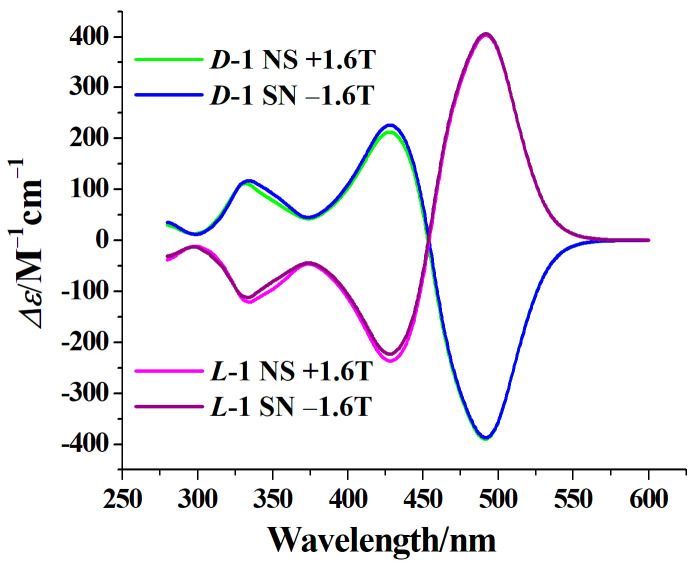
CD spectra of *L*-**1** and *D*-**1** in DMF solution under an NS (+1.6 T) or SN (−1.6 T) field (c = 0.2 gL^−1^; optical path = 5 mm).

To study the magneto-optical properties of *L*-**1** and *D*-**1**, we further measured their CD spectra when a magnetic field (±1.6 T) was applied (Figure 5). It should be noted that the positive (+1.6 T, NS) and negative magnetic fields (−1.6 T, SN) refer to the parallel and antiparallel direction of the magnetic field to the polarized light, respectively. As shown in Figure 5, the CD spectra of *L*-**1** and *D*-**1** under the ±1.6 T magnetic field also have mirror symmetry: the *D*-**1** (NS, +1.6 T) curve is mirror symmetrical with *L*-**1** (SN, −1.6 T), while the *D*-**1** (SN, −1.6 T) curve is mirror symmetrical with *L*-**1** (NS, +1.6 T). Notably, the positions of the Cotton peaks of the CD spectra of *L*-**1** and *D*-**1** at ±1.6 T are similar to those of their CD spectra at 0 T. The pure MCD signals *L*-**1** and *D*-**1** could be obtained based on MCD = [CD(NS) − CD(SN)]/2/M (here M = 1.6 T) [38,40,41,44,45]. The pure MCD spectra show strong troughs at 350 nm and 437 nm for *L*-**1** and at 350 nm and 437 nm for *D*-**1** (Figure S5); these two troughs for each isomer are ascribed to the π-π* and n-π* transitions of the C=N chromophore, respectively [42,45,46].

The g_MCD_ values of *L*-**1** and *D*-**1** were then calculated based on *g*_MCD_ = 2(ε_+_(*B*) − ε_+_(−*B*))/(ε_+_(*B*) + ε_+_(−*B*)) [56] because the |g_max(MCD)_| value can be used to estimate the strength of the magneto-optical Faraday effect [56]. As shown in Figure S6, the |g_max(MCD)_| values of *L*-**1** and *D*-**1** at room temperature are 0.026 T^−1^ and 0.027 T^−1^, respectively; these values are comparable with those of the homochiral 3d–4f complexes [R,R-/S,S-ZnLDy(H_2_O)(NO_3_)_3_] {H_2_L = 2-((E)-((1R,2R)/(1S,2S)-2-((E)-2-hydroxy-3-methoxybenzylideneamino)-1,2-diphenylethylimino)methyl)-6-methoxyphenol} (0.067 T^–1^) [46] and [Mn_10_Ln_6_(R-/S-L_1_)_6_(L_2_)_2_(Ac)_12_(μ_5_-O)(μ_4_-O)_6_(μ_3_-OH)_6_(μ_2_-H_2_O)_2_][Mn_6_Ln_2_(R-/S-L_1_)_6_(L_2_)_2_(Ac)_2_(μ_4_-O)_2_(μ_3_-OH)_2_(H_2_O)_2_]·4ClO_4_·10H_2_O·CH_3_CH_2_OH {R-/S-H_2_L = (R)-/(S)-2-(((1-hydroxybutan-2-yl)imino)methyl)-5-methoxyphenol; HL_2_ = 2-hydroxy-4-methoxybenzaldehyde} (0.012 T^–1^) [44], but are obviously smaller than those of 4f SMMs based on homochiral β-diketone ligands (≥0.34 T^–1^) [40,41,42] and Cu-Dy zero-field SMMs (≥0.44 T^–1^) [39,45]. This may be related to the fact that the SMM properties of *L*-**1** and *D*-**1** are not as good as the latter.

### 2.4. Nonlinear Optical Properties

**Figure 6:**
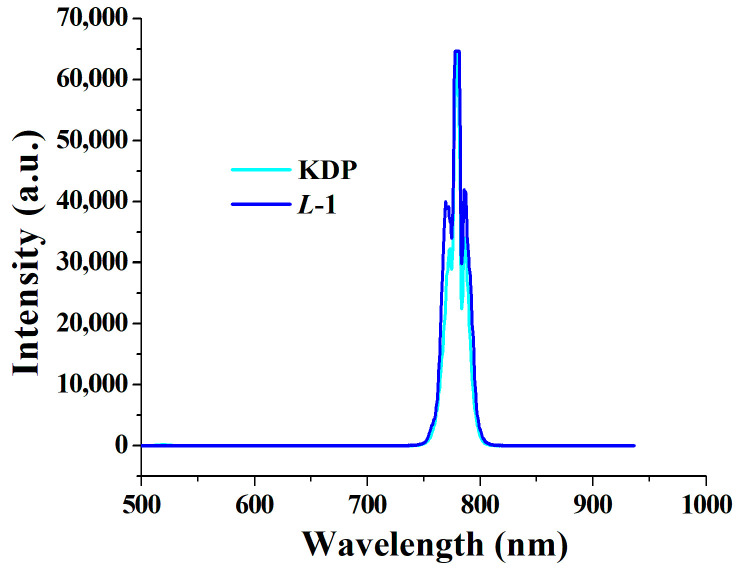
SHG spectra of crystalline samples of *L*-**1** and KDP under excitation at λ = 1550 nm (T_int =_ 0.5 s).

Since *L*-**1**/*D*-**1** is a chiral crystalline complex, we investigated its second-order nonlinear optical properties. The second harmonic generation (SHG) efficiency of *L*-**1** is as strong as that of the reference sample KDP (KH_2_PO_4_) (Figure 6), and the SHG intensity of *L*-**1** (1.0 × KDP) is equal to that of [DyZn_2_(S,S-L_Schiff_)Cl_2_(H_2_O)][DyZn_2_(S,S-L_Schiff_)Cl_2_(MeOH)](ClO_4_)_2_·MeOH·0.5H_2_O (1.0 × KDP) and [DyZn_2_(S,S-L_Schiff_)Br_2_(H_2_O)][DyZn_2_(S,S-L_Schiff_)Br_2_(MeOH)](CF_3_SO_3_)_2_ (1.0 × KDP) [57], but is obviously larger than those of chiral Dy(III) SMMs (0.7 × KDP) [58] and other chiral Zn-Ln SMMs (≤0.2 × KDP) [26,59,60]. Therefore, *L*-**1**/*D*-**1** is a potential second-order nonlinear optical molecular material. By the way, simultaneous measurements showed that *L*-**1**/*D*-**1** does not show any third-order nonlinear optical properties, which may be due to the lack of a strong electronic push–pull group in the ligand of *L*-**1**.

## 3. Experimental Section

All chemicals were analytically pure, purchased from commercial suppliers, and ready for use.

### 3.1. Materials and Instruments

The elemental analyses (C, H, N) were determined using a Thermo Flash Smart (Thermo Fisher Scientific, Waltham, MA, USA) elemental analyzer. The infrared spectra were recorded on a Bruker VERTEX 70v spectrophotometer (BRUKER OPTICS, Karlsruhe, Germany) using pressed KBr pellets. The UV-vis spectrum of H_2_L_Schiff_ was measured on a UH5700 Spectrophotometer (Hitachi hi-tech Co., Ltd., Tokyo, Japan). The circular dichroism (CD) spectra were recorded on a JASCO J-1700 spectropolarimeter (JASCO, Tsukuba, Japan) at room temperature with a 5 mm optical path, and a permanent magnet (+1.6 or −1.6 T) was used to measure MCD spectra. Magnetic properties were recorded on a Quantum Design MPMS-XL5 (SQUID) magnetometer (Quantum Design, San Diego, CA, USA). The diamagnetism of the molecule was corrected with Pascal’s constant.

### 3.2. Nonlinear Optical (NLO) Response Measurements

An ultrafast fiber laser (NPI Lasers, Rainbow 1550 OEM) (PL OPTICS, Nanjing, China) was utilized as the excitation source to output 100 fs pulses at 1550 nm with a repetition rate of 80 MHz. This laser beam was then focused by an aspheric lens with a numerical aperture of 0.8, forming a laser spot with a beam waist radius of 2 μm. The spectra of the second-harmonic generation (SHG) and the third-harmonic generation (THG) were obtained using a cooled fiber optic spectrometer (Ideaoptics, NOVA, Shanghai, China). For comparison, the same integration time (T_int_ = 0.5 s) was used to obtain SHG and THG signals for the sample and reference material. The SHG and THG mappings were monitored by integrating SHG and THG spectra at different locations over an integration range of 500–550 nm and 750–800 nm for THG and SHG, respectively.

### 3.3. Synthesis of H_2_L_Schiff_

1-Hydroxy-2-naphthaldehyde (10 mmol) and pyrazinoic acid hydrazide (10 mmol) were added to 30 mL of methanol; the reaction mixture was refluxed for 3 h and then cooled to room temperature. The resulting light-yellow precipitate was collected by filtration and washed with 3 × 10 mL of ethanol before being dried in air. The yield was about 90%. The analytical calculations for H_2_L_Schiff_ (C_16_H_12_N_4_O_2_, %) are as follows: C, 65.75; H, 4.14; N, 19.17%; found (%): C, 65.56; H, 4.18; N, 19.07%. The FT-IR peaks (KBr, cm^−1^) for H_2_L_Schiff_ are as follows: 3315 (m), 3052 (w), 1690 (s), 1635 (w), 1600 (m), 1565 (w), 1523 (m), 1502 (w), 1463 (w), 1402 (w), 1358 (w), 1315 (m), 1265 (w), 1227 (w), 1160 (m), 1146 (w), 1109 (w), 1095 (w), 1019 (m), 988 (w), 958 (w), 903 (w), 877 (w), 849 (w), 810 (m), 786 (w), 763 (w), 746 (w), 678 (w), 555 (m), 480 (w), 444 (w), and 427 (w).

### 3.4. Syntheses of L-***1*** and D-***1***

A mixture of H_2_L_Schiff_ (0.25 mmol), *L*/*D*-proline (0.25 mmol), Dy (CF_3_SO_3_)_3_ (0.25 mmol), and LiOH·H_2_O (0.75 mmol) in 5 mL of DMA was stirred for 24 h to form a brownish-red solution, which was filtered and transferred to a beaker. By slowly evaporating the solvent, single red crystals of *L*-**1** and *D*-**1** appeared a few weeks later. The yield was as follows: about 20% based on Dy. The analytical calculations for *L*-**1** (C_147_H_153_Dy_6_N_35_O_34_, %) are as follows: C, 44.94; H, 3.93; N, 12.48%; found (%): C, 44.89; H, 3.97; N, 12.45%. The FT-IR peaks (KBr, cm^−1^) for *L*-**1** are as follows: 3434 (s, br), 3044 (w), 2935 (w), 2874 (w), 1595 (s), 1555 (m), 1534 (m), 1516 (w), 1497 (w), 1472 (m), 1433 (m), 1405 (m), 1327 (m), 1239 (w), 1201 (w), 1176 (w), 1151 (w), 1102 (w), 1056 (w), 1033 (w), 980 (w), 926 (w), 889 (w), 860 (w), 794 (w), 770 (w), 747 (w), 704 (w), 688 (w), 619 (w), 583 (w), 566 (w), 509 (w), 476 (w), and 429 (w). The analytical calculations for *D*-**1** (C_147_H_153_Dy_6_N_35_O_34_, %) are as follows: C, 44.94; H, 3.93; N, 12.48%; found (%): C, 44.98; H, 3.97; N, 12.44%. The FT-IR peaks (KBr, cm^−1^) for *D*-**1** are as follows: 3434 (s, br), 3044 (w), 2938 (w), 2875 (w), 1594 (s), 1555 (m), 1534 (m), 1516 (w), 1497 (w), 1472 (m), 1433 (m), 1406 (m), 1327 (m), 1238 (w), 1201 (w), 1176 (w), 1151 (w), 1102 (w), 1056 (w), 1034 (w), 980 (w), 926 (w), 889 (w), 860 (w), 794 (w), 771 (w), 748 (w), 704 (w), 688 (w), 619 (w), 583 (w), 566 (w), 509 (w), 476 (w), and 429 (w).

### 3.5. X-ray Single-Crystal Structure Measurement

Single-crystal diffraction data for *L*-**1** and *D*-**1** were collected on a Rigaku MM007HF (RIGAKU, Tokyo, Japan) diffractometer () with graphite-monochromated Mo-Kα radiation (λ = 0.71073 Å) at 173 K. The two structures were solved using the olex2.solve structure solution program and refined using the ShelXL-2015 refinement package. The squeeze technology was adopted to refine these two structures since the lattice solvent molecules DMA and H_2_O in *L*-**1** and *D*-**1** were disordered. Notably, the pyrrolidine ring in *L*/*D*-proline ligands was disordered in two positions and idealized by bond distances. All non-hydrogen atoms were refined anisotropically, and all hydrogen atoms were allowed for as riding atoms. The crystallographic data and refinement parameters are listed in Table 1.

## 4. Conclusions

In summary, a pair of enantiomers with the novel triangular Dy_6_ core was prepared using the new hydrazone Schiff base bridging ligand (E)-N′-((1-hydroxynaphthalen-2-yl)methylene)pyrazine-2-carbohydrazide and the chiral ligand *L*/*D*-proline. During the reaction, CO_2_ in the air was fixed to form the carbonato ligand, which plays a critical role in the construction of the homochiral Dy_6_ complexes as a central bridging ligand. This pair of homochiral Dy_6_ clusters exhibited intramolecular ferromagnetic coupling and a frequency-dependent AC magnetic susceptibility and displayed a distinct magneto-optical Faraday effect and an SHG response as strong as KDP. This study demonstrates that the fixation of CO_2_ in the air can be used to construct chiral cluster complexes, which provides a unique synthetic pathway for multifunctional molecular materials.

## Figures and Tables

**Table 1:** Crystal data and structural refinement parameters for *L*-**1** and *D*-**1**.

	*L-***1** (Squeeze)	*D-***1** (Squeeze)
formula	C_127_H_104_Dy_6_N_30_O_27_	C_127_H_104_Dy_6_N_30_O_27_
*F_W_*	3457.40	3457.40
crystal system	monoclinic	monoclinic
space group	*C2*	*C2*
*a* [Å]	33.8797(5)	33.8283(9)
*b* [Å]	17.3214(2)	17.3710(4)
*c* [Å]	26.3394(3)	26.3514(6)
*β* [°]	102.5770(10)	102.613(2)
*V* [Å^3^]	15,086.2(3)	15,111.2(6)
*Z*	4	4
*ρ*_calc_ [g·cm^−3^]	1.522	1.520
*μ* [mm^−1^]	3.006	3.002
*T* [K]	170	170
*λ* [Å]	0.71073	0.71073
reflections collected	142,419	132,511
unique reflections	41,244	39,636
observed reflections	33,159	29,637
parameters	1987	1981
GoF [*I* ≥ 2*σ* (*I*)]	1.048	1.063
*R*_1_ [*I* ≥ 2*σ* (*I*)]	0.0568	0.0602
*_W_R*_2_ [*I* ≥ 2*σ* (*I*)]	0.1380	0.1439
Flack parameter	0.086(12)	0.005(7)
CCDC number	2,363,753	2,363,754

## Data Availability

Data are contained within the article and Supplementary Materials.

## Supplementary Materials

The following supporting information can be downloaded at https://www.mdpi.com/article/10.3390/molecules29143402/s1: X-ray crystallographic data file in CIF format, CCDC number 2,363,753 for *L*-**1** and 2,363,754 for *D*-**1**; Tables S1 and S2: Dy(III) ion geometry analysis for *L*-**1** using SHAPE 2.1 software; Figure S1: M vs. H/T plots of *L*-**1** at 2–6 K; Figure S2: plots of χ″ vs. T for *L*-**1** at 1399 Hz (H_dc_ = 0 Oe and 2000 Oe); Figure S3: Debye plots of *L*-**1** for the indicated AC frequencies; Figure S4: UV-vis spectra of H_2_L_Schiff_ in DMF solution; Figure S5: MCD spectra of *L*-**1** and *D*-**1**; Figure S6: g_MCD_ of *L*-**1** and *D*-**1**.
