# Supplementary material for: Assembly of Homochiral Magneto-Optical Dy6 Triangular Clusters by Fixing Carbon Dioxide in the Air
Source: Molecules. 2024 Jul 19;29(14):3402. doi: 10.3390/molecules29143402 (PMC11279596; doi:10.3390/molecules29143402)
Supplement: Supplementary file 1 [file molecules-29-03402-s001.zip › molecules-3098657-supplementary.pdf]

Supporting Information

Assembly of Homochiral Magneto-Optical Dy6 Triangular Clusters by Fixing Carbon Dioxide in the Air

Cai-Ming Liu [1,2,*], Xiang Hao [1] and Xi-Li Li [3]

[1] Beijing National Laboratory for Molecular Sciences, CAS Key Laboratory of Organic Solids, Institute of Chemistry, Chinese Academy of Sciences, Beijing 100190, China; haoxiang@iccas.ac.cn
[2] School of Chemical Sciences, University of Chinese Academy of Sciences, Beijing 100049, China
[3] Henan Provincial Key Laboratory of Surface and Interface Science, Zhengzhou University of Light Industry, Zhengzhou 450002, China; lixl@zzuli.edu.cn
* Correspondence: cmliu@iccas.ac.cn

Contents

1. Tables S1 and S2, continuous Shape Measures calculation for Dy atoms in ***L*-1**.
2. Figure S1, *M versus H*/*T* plots at 2-6 K of *L*-**1**.
3. Figure S2. Plots of χ'' *versus T* at 1399 Hz for *L*-**1** (H_{dc} = 0 Oe and 2000 Oe).
4. Figure S3. Debye plots of *L*-**1** for the indicated ac frequencies. The solid lines represent the fit of the data by applying Equation (1).
5. Figure S4. UV-vis spectra of H_2L_{Schiff} in DMF solution (*c* = 0.04 gL^{-1}).
6. Figure S5, MCD spectra of *L*-**1** and *D*-**1**.
7. Figure S6. g_{MCD} of *L*-**1** and *D*-**2**.

Table S1. 10-coordinated Dy (III) ion geometry analysis by SHAPE 2.1 software for *L*-**1**.

Configuration	ABOXIY Dy2	ABOXIY Dy3	ABOXIY Dy5
Decagon (D_{10h})	33.225	34.012	33.651
Enneagonal pyramid (C_{9v})	22.301	22.581	22.420
Octagonal bipyramid (D_{8h})	18.199	17.952	18.168
Pentagonal prism (D_{5h})	12.020	11.698	11.350
Pentagonal antiprism (D_{5d})	13.258	13.183	13.239
Bicapped cube J15 (D_{4h})	10.881	11.230	11.075
Bicapped square antiprism J17 (D_{4d})	**2.886**	**3.156**	**3.052**
Metabidiminished icosahedron J62 (C_{2v})	8.415	8.571	8.468
Augmented tridiminished icosahedron J64 (C_{3v})	16.571	16.403	16.383
Sphenocorona J87 (C_{2v})	3.669	3.450	3.798
Staggered Dodecahedron (2:6:2) (D_2)	4.933	4.746	4.756
Tetradecahedron (2:6:2) (C_{2v})	4.017	3.797	3.894
Hexadecahedron (2:6:2) or (1:4:4:1) (D_{4h})	9.666	9.820	9.728

Table S2. 8-coordinated Dy (III) ion geometry analysis by SHAPE 2.1 software for *L*-**1**.

Configuration	ABOXIY Dy1	ABOXIY Dy4	ABOXIY Dy6
Octagon(D_{8h})	30.155	30.637	28.375
Heptagonal pyramid(C_{7v})	24.551	24.974	24.696
Cube (O_h)	12.455	11.673	13.354
Hexagonal bipyramid(D_{6h})	8.221	7.940	9.478
Square antiprism (D_{4d})	3.004	3.505	3.297
Triangular dodecahedron (D_{2d})	3.379	3.639	3.460
Johnson gyrobifastigium J26 (D_{2d})	10.452	9.519	10.492
Johnson elongated triangular bipyramid J14 (D_{3h})	25.227	25.019	25.157
Biaugmented trigonal prism J50 (C_{2v})	**2.249**	**2.679**	2.218
Biaugmented trigonal prism (C_{2v})	2.474	2.955	**2.118**
Snub diphenoid J84 (D_{2d})	3.387	3.659	3.598
Triakis tetrahedron (T_d)	8.233	8.055	9.597
Elongated trigonal bipyramid (D_{3h})	22.705	22.187	21.718

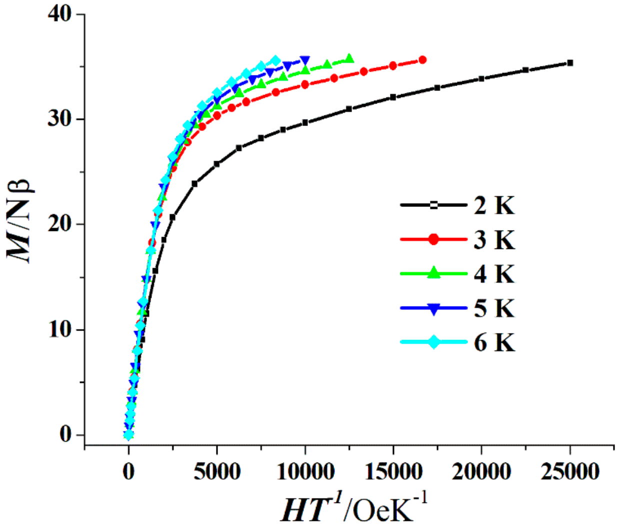

Figure S1. *M versus H*/*T* plots at 2-6 K of *L*-**1**.

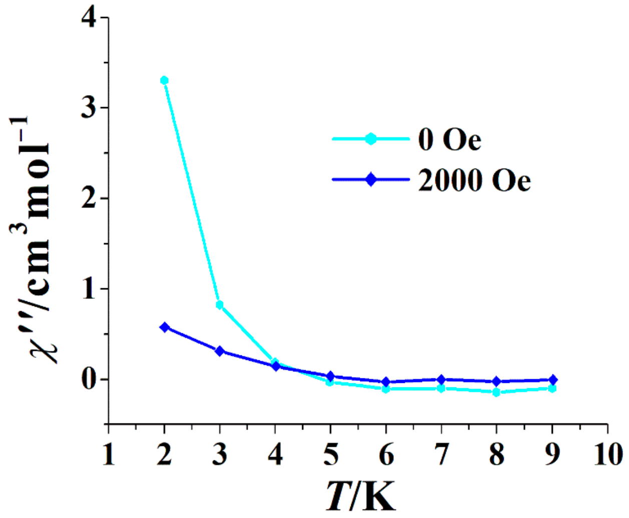

Figure S2. Plots of χ'' *versus T* at 1399 Hz for *L*-**1** (H_{dc} = 0 Oe and 2000 Oe).

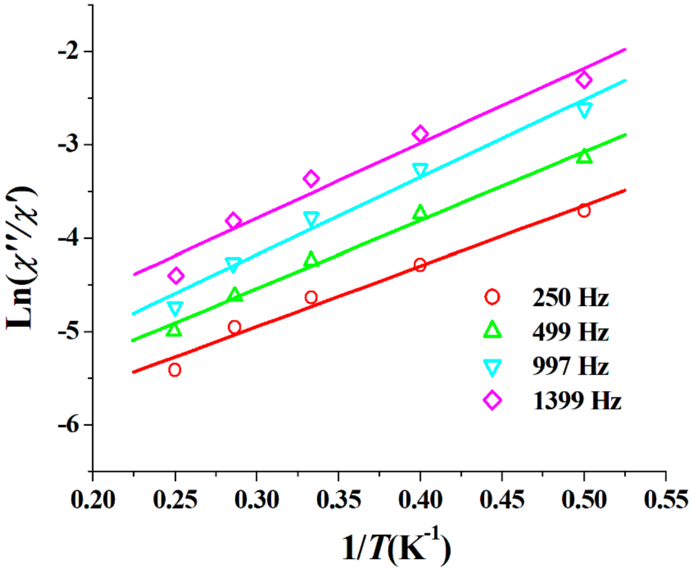

Figure S3. Debye plots of *L*-**1** for the indicated ac frequencies. The solid lines represent the fit of the data by applying Equation (1).

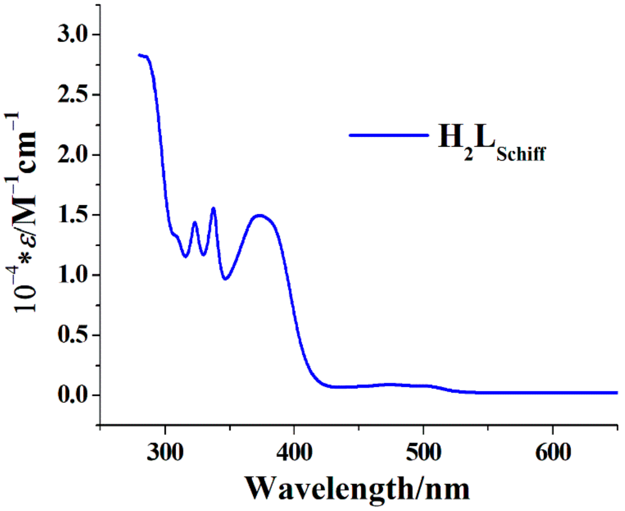

Figure S4. UV-vis spectra of H_2L_{Schiff} in DMF solution (c = 0.04 gL^{-1}).

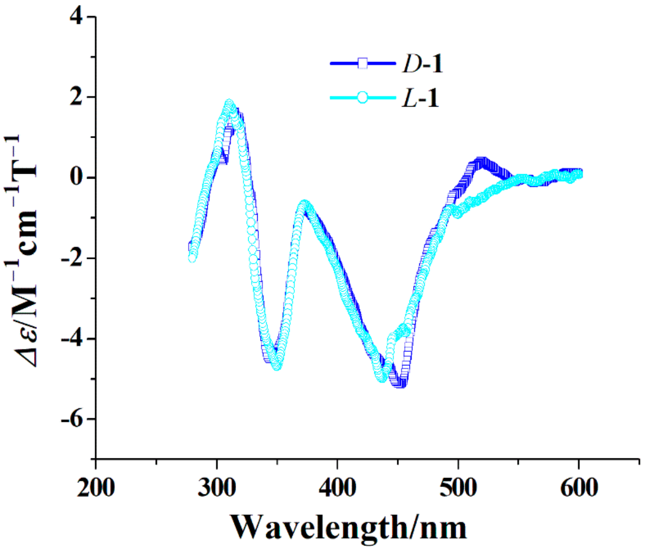

Figure S5. MCD spectra of *L*-**1** and *D*-**1**.

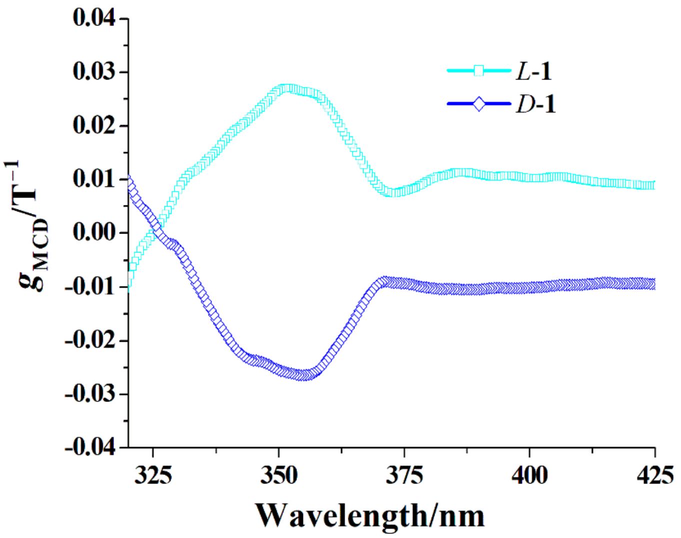

Figure S6. g_{MCD} of *L*-**1** and *D*-**1**.
